# Green synthesis of TiO_2_ for furfural production by photohydrolysis of tortilla manufacturing waste

**DOI:** 10.1038/s41598-023-41529-z

**Published:** 2023-09-16

**Authors:** Janneth López-Mercado, Martha-Isabel González-Domínguez, Francisco-Javier Reynoso-Marin, Brenda Acosta, Elena Smolentseva, Apolo Nambo

**Affiliations:** 1 Ingeniería en Nanotecnología, Universidad de La Ciénega del Estado de Michoacán de Ocampo, 59103 Sahuayo, Mexico; 2 Cátedras CONACYT, Coordinación para la Innovación y la Aplicación de la Ciencia y la Tecnología, Universidad Autónoma de San Luis Potosí, 78000 San Luis Potosí, Mexico; 3 Centro de Nanociencias y Nanotecnología, Universidad Nacional Autónoma de México, Km. 107 Carretera Tijuana a Ensenada, C.P. 22860 Ensenada, Baja California Mexico; 4 Conn Center for Renewable Energy Research, University of Louisville, Louisville, 40292 USA

**Keywords:** Biochemistry, Biotechnology, Chemical biology, Environmental sciences, Chemistry, Engineering, Materials science, Nanoscience and technology

## Abstract

Corn nixtamalization generates a waste byproduct that requires diverse environmental preservation measures depending on the country. Such measures could include catalytic and advanced oxidation processes. This study aims to exploit the hemicellulose within the nejayote (32.5%) to create added value chemicals such as furfural using photocatalytic hydrolysis. In the present work, titania (TiO_2_) nanoparticles (NPs) were greenly synthesized using *Ricinus Communis* (RC), *Moringa Oleifera* (MO) or *Bougainvillea Spectabilis* (BS) plant extracts. Obtained nanoparticles were characterized using XRD, SEM, EDS, BET, XPS and UV–vis techniques. Furthermore, the photocatalytic performance of the obtained samples was evaluated in the furfural production from nejayote. Furfural yield reached 44% in 30 min using the BS synthesized material, which is 1.6 × the yield obtained by the material synthesized with MO extract (26.4% at 45 min) and 6 × the yield obtained by the material obtained with RC (7.2% at 90 min). Such results have not been reported before in the literature and could be the groundwork for novel waste treatments in the tortilla-making industry.

## Introduction

Consumption of proceed food has increased dramatically in recent years growing agro-industrial waste generation, which is considered an environmental, social and economic problem worldwide. Tortilla making industry is a wide spread business with huge success in México, Central America, United States of America, Asia and Europe. The tortilla-making involves a pretreatment step for the corn called nixtamilization^1,2^. Nixtamilization generates large amounts of alkaline liquid waste, known as nejayote, which are usually disposed of in the urban sewer system. Nejayote contains significant amounts of organic matter (carbohydrates, proteins, phenolic compounds, among others) and inorganic compounds (calcium and magnesium from the lime used in the process)^2,3^. Nejayote is considered to be an environmental pollutant that requires processing, One of the processing technologies that have been proposed is the work reported by Castro-Muñoz et al. that use nejayote to obtain carbohydrates, calcium and phenolic compounds by means of a filtering membrane^4^. Ramírez-Romero et al. and López-Pacheco et al. use nejayote as a culture medium to obtain by-products of interest, such as: probiotics and bateriocins^5,6^. However, it could also be considered as a source of biocomposites and could be converted to value-added chemicals, as has been reported with other wastes that are rich in polysaccharides (cellulose and hemicellulose)^7,8^.

Processes for the transformation of liquid wastes containing polysaccharides, such as nejayote^6^, to compounds with high added value can utilize hydrolysis or photocatalysis reactions. Dumesic's team has used hydrolysis for the conversion of corn waste, using high temperature and high pressure conditions to obtain furfural or hydroxymethyl furfural^9^. The photocatalysis reaction has not been extensively studied for this purpose; however, there is a large amount of literature for the processing of wastewater with high organic loads^10–14^. Photocatalysis is a process that requires the presence of UV–Vis radiation, environmental conditions (in some studies), and a photocatalytic medium achieving partial oxidation (obtaining value-added chemicals) or total oxidation (water and CO_2_). Some materials that have been used in photocatalysis are based on TiO_2_ using several morphologies and formulations: TiO_2_ nanoparticles in their anatase crystalline phase^10,11,14^, Ag/TiNPs^15^, O_2_/Ag^16,17^, among others. The efficiency of TiO_2_ is attributed to its optoelectronic and physicochemical properties^16,17^, which depend directly on the conditions and methods of synthesis used. TiO_2_ NPs are synthesized through various methodologies^18–20^. Among the most recognized are sol–gel^11,21^, hydrothermal^22,23^, combustion^24^, vapor phase^25,26^, controlled precipitation, polymer precursor^27^, etc. However, the application of such methodologies requires the use of solvents, reducing agents and stabilizing materials, which can generate various toxic or harmful compounds; moreover, it increases the production costs of TiO_2_ NPs. Green synthesis may be considered as an alternative way to reduce health risks and the environmental impact during the synthesis of nanomaterials^28^. This methodology explores the potential of certain plant extracts known as S-Met (terpenes, essential oils, flavonoids, phenols, etc.), to reduce metal ions and to generate stable nanoparticles with unique morphologies^13^. Until now, green synthesis has been mainly applied to produce noble metals (Ag, Au, Pt and Pd, Cu) and metal oxides (Zn and Ti) NPs^29,30^. The source of S-Met can be associated with the demand for specific compounds (polyphenols, flavonoids, acids, etc.) required for the synthesis of NPs and their final application, as well as the geographical conditions and variety of available plants. For example, plants of historical relevance for disease treatment (Indian rosewood, gooseberry, tea leaves, etc.) are widely studied for the synthesis of metal NPs with antimicrobial applications^30^. Some researchers reported the use of S-Met, extracted from BS, MO and RC, for the synthesis of silver NPs with various applications^31,32^. Recently, anatase TiO_2_ NPs have been synthesized using alcohol extracts from *B. spectabilis,* obtaining materials with crystal sizes of about 6 nm; other TiO_2_ NPs synthesized from *R. communis* extracts have drawn some attention since the extracts contain catechins, flavonols, hydroxycoumarins, and scopoletin all of which contain OH groups that result in more photoactive materials^33^.

The present work aims to establish the basis for photocatalytic processes capable of transforming the organic content of nejayote (polyacharides) into furfural using greenly synthesized materials. The TiO_2_ NPs obtained through the green synthesis using S-Met extracts from RC, MO and BS plants from the marshland region of Chapala, México are analyzed. The physicochemical and electronic properties of the greenly synthesized TiO_2_ NPs are discussed, as well as their potential use for furfural production by photohydrolysis of the unexplored nejayote. Commercial TiO_2_ (comm TiO_2_) was also analyzed parallelly and presented as reference material.

## Materials and methods

### Materials

Sulfuric acid (H_2_SO_4_ Sigma Aldrich), sodium bicarbonate (NaHCO_3_, Sigma Aldrich), hydrochloric acid (HCl Sigma Aldrich), magnesium (Mg, Golden Bell), titanium isopropoxide (Ti[OCH(CH_3_)_2_]_4_, Sigma Aldrich) and comm TiO_2_ (TiO_2_, Fermont) were used as reagents for the analysis of S-Met and TiO_2_ NPs synthesis. The furfural (C_4_H_3_OCHO, Sigma Aldrich) was used as the standard for the calibration curve. The RM, MO and BS plants were collected nearest Chapala Lake, in Jalisco, México. For the photohydrolysis reaction, the nejayote sample was obtained from a local industry in Sahuayo, Michoacán, México. Nejayote is considered fresh and useful up to 3 days before its collection.

### Metabolites extraction

Different sections of each plant were used for the S-Met extraction, flowers and bracts from BS, and leaves from RC and MO. The methodology commonly applied to extract the S-Met is reported^31^. In the present work, the extraction conditions were modified to increase the efficiency of the process. In brief, each plant extract was obtained by mixing 20 g of the plant with 100 mL of water. Then, the mixture was heated up to 90 °C and kept for 10 min. Finally, the aqueous extract was separated by vacuum filtration and kept refrigerated until use^34^. All methods were performed in accordance with the relevant guidelines and regulations.

### Qualitative phytochemical analysis of extracts

Each plant extract underwent a phytochemical screening to know the phytoconstituents of the obtained S-Met. The presence of flavonoids, tannins, phenols, quinones and saponins was evaluated following well-known methodologies such as Shinoda, Foam tests, FeCl_3_ test and others, described^35–37^.

### Nanoparticles green synthesis

Titanium isopropoxide (12 mL) was mixed with 78 mL of distilled water and 10 mL of the S-Met plant extract obtained from each selected plant. The formed suspension was kept at 50 °C for 4 h under magnetic stirring. Subsequently, the obtained product was washed with distilled water and dried at 60 °C overnight. Finally, the samples were calcined at 500 °C for 3 h. The powdered sample was collected and saved for later testing and characterization.

### Physicochemical characterization

The morphology and elemental composition of the greenly synthesized TiO_2_ NPs were studied by scanning electron micrographs and X-Ray energy dispersion spectra, respectively, obtained with a JSM-6610-LV microscope, JEOL. Structural analysis of samples was performed using X-ray diffraction patterns collected in a D8 Avance A25 Bruker X-Ray diffractometer equipped with CuKα radiation. Textural properties of titania samples were determined by the N_2_ thermal adsorption measurements using TriStar II-3020 equipment, Micromeritics. Before the analysis, the samples were dried under vacuum (10^–3^ torr) at 300 °C for 3 h using a VacPrep 061-Sample degas system, Micromeritics. Obtained isotherms were analyzed via the Brunauer-Emmet-Teller (BET) and Barrett-Joyner-Halenda (BJH) models for the surface area and porosity determination, respectively. The optical characterization of the powder samples was determined by UV–Vis spectroscopy in diffuse reflectance mode using an UV-3600 Plus UV–VIS-NIR spectrophotometer, Shimadzu, equipped with an integrating sphere. The samples were placed in a quartz cell with 2 mm in the light path for the measurement. The electronic state of prepared samples was studied by the XPS technique with a PHOIBOS spectrometer, SPECS, with 150 WAL hemispherical energy analyzer and a monochromatic source (AlKα Xray, 1486.6 eV).

### Photocatalytic evaluation

#### Nejayote characterization

Fresh nejayote (100 mL) was treated with 4%w of H_2_SO_4_ at 200 °C for 2 h, using an autoclave. Then, the obtained solution/suspension was analyzed by means of liquid chromatography using an Ultimate 300 High-Pressure Liquid Chromatographer, Thermo Scientific, equipment to determine the presence of sugars. Additionally, 100 mL of fresh nejayote was diluted in 100 mL of water and then centrifuged. The supernatant was separated while the precipitated solids were calcined at 500 °C for 5 h. Calcined samples were analyzed by EDS to determine the inorganic matter.

#### Nejayote photohydrolysis

The photocatalytic performance of the obtained TiO_2_ NPs was evaluated at the photohydrolysis of nejayote to produce furfural. Reactions were carried out in a cylindrical quartz batch reactor of 1 L with a constant oxygen flow rate of 2 ml per minute. The reactor had a continuous stirring speed of 400 rpm at room temperature. Typically, 1 g of catalyst was dispersed in 800 mL of nejayote (fresh sample without any variations in the nixtamilization process). Before the light irradiation, the reaction mixture was stirred for 30 min to reach adsorption equilibrium. Then, the UV-light lamp with a wavelength of 365 nm was turned on and the reaction progress was monitored by analyzing the products at 5, 15, 30, 45, 60, and 90 min. The reaction mixture samples were centrifuged for 5 min at 700 rpm and the catalyst was separated and stored. The furfural concentration was quantified using Perkin Elmer Clarus 680 gas chromatograph with a mass spectrometer (GC–MS), and the column employed was a 30 m capillary column Elite 1 PerkinElmer (inner diameter of 0.32 mm and film thickness of 0.25 μm) with a flow of 1.5 ml/s of Helium as carrier gas. All reactions were performed in triplicate.

For the catalyst recycle evaluation, the recovered catalyst from BS extract was calcined at 500 °C for 2 h with a heating rate of 10 °C per min. The calcined material was characterized using XRD, SEM–EDS, and UV–Vis.

## Results and discussion

### Metabolites characterization

It is well-known that S-Met assists the biosynthesis of metal NPs by acting as a reducing and/or stabilizing agent^38^. The qualitative information on phytochemical constituents of obtained S-Met used in the green synthesis of TiO_2_ NPs is shown in Table 1. The presence of flavonoids, saponins, tannins and phenols characterized the S-Met extracts from the three plants. The absence of essential oils and fatty substances was observed for all analyzed plants; meanwhile, flavones were only found in RS and MO extracts. The slight variations in the constituents found for each plant may be attributed to using different plant parts for the extraction: leaves from RC and MO where used, while flowers and branches from BS were used^38^. Flavonoids and phenols are considered potential reducers of metallic precursors^39^. Indeed, Bharathi^31^ showed that flavonoids and phenols obtained from BS are the principal for forming silver nanoparticles by reducing silver nitrate. On the other hand, Mintiwab and Jeyaramraja reported that flavonoids, tannins, saponins, alkaloids, triterpenes and steroids extracted from RC leaves allow for the reduction and stabilization of silver nanoparticles^39^. Moodley reported that flavones, terpenoids and polysaccharides obtained from MO leaves are primarily responsible for the reduction and stabilization of silver^32^ and TiO_2_ NPs^40^. It is expected that found constituents (flavonoids, saponins, tannins and phenols) can reduce the titanium precursor salt to titanium ion intermediates for their further conversion into TiO_2_ NPs.

**Table 1:** Qualitative information on phytochemical constituents of secondary metabolites.

Method	Phytochemical constituent	*Bougainvillea spectabilis*	*Moringa oleífera*	*Ricinus communis*
Shinoda test	Flavonoids	+	+	+
Shinoda test with heat	Flavonoids	+	+	+
H_2_SO_4_ test	Flavone	−	+	+
	Quinones	+	−	−
Foam test (5 min)	Saponins	+	+	+
Foam test (30 min)	Saponins	+	+	+
NaHCO_3_ test	Saponins	+	+	+
HCl test	Tannins and phenols	+	+	+
FeCl_3_ test	Tannins and phenols	+	+	+
Essential oils and fatty substances	Essential oils and fatty substances	−	−	−

− Absent, + Present.

### Nanoparticles characterization

The elemental analysis of TiO_2_ samples confirmed the presence of titanium and oxygen mostly (Table S1). However, some traces of potassium were found in the samples obtained with S-Met plants extract. The latter may be assigned to the chemical composition of plants, where inorganic matter, such as potassium, indicates that the extracellular inorganic moieties are absorbed in the TiO_2_ NPs surface^40,41^. Nevertheless, its contribution was insignificant to interfere with the sample properties, such as the morphology or composition.

Figure S1 presents the typical SEM micrographs of the TiO_2_ NPs samples prepared from different plant extracts, with comm TiO_2_ as reference. The green synthesis approach resulted in a quasispherical shape of TiO_2_ NPs with a size below 100 nm. In comparison, comm TiO_2_ was characterized with larger aggregates (up to 300 nm) (Fig. S1). According to the micrographs analysis, the diameters of the formed TiO_2_ NPs were 49, 41 and 55 nm when BS, MO and RC were used, respectively. Despite the slight differences in the qualitative characterization of the S-Met extract (see Table 1), it seems that both the S-Met dispersion and solubility in the reaction media are responsible for the morphology inhomogeneity of the obtained TiO_2_ NPs, as was discussed for the Ag nanoparticles synthesis from BS and MO extracts^31,32^. Indeed, some studies related to the green synthesis of TiO_2_ NPs, using the same Ti precursor (Ti[OCH(CH_3_)_2_]_4_), resulted in the formation of heterogeneous morphology accompanied by the contribution of NPs with different sizes (even up to micrometers)^42^, unless a tensoactive is used^43^.

**Figure 1:**
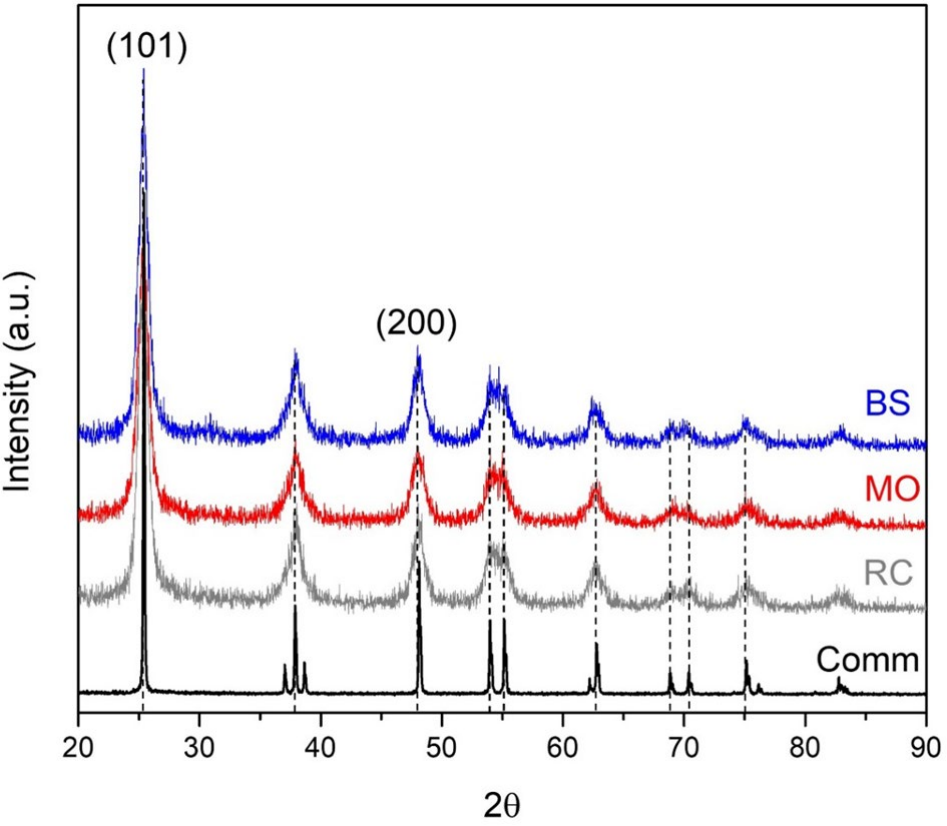
X-ray diffraction patterns of TiO_2_ samples prepared under a green synthesis from different plant extracts: *Ricinus communis* (RC)*, Moringa oleífera* (MO) and *Bougainvillea spectabilis* (BS). A diffractogram from commercial TiO_2_ (Comm) is presented as a reference. Bragg index corresponds to the PDF 00–064-0863 chart.

Figure 1 shows the X-ray diffraction patterns for the greenly synthesized samples compared to that for the reference comm TiO_2_ powder. All samples were characterized with well-distinguishable reflections between 20° and 90° of 2θ. However, the reflections for TiO_2_ prepared by green synthesis were less intensive and broader than those of the commercial sample. XRD analysis revealed the preferential formation of the TiO_2_ anatase phase. The most intensive peaks were located at 25° and 48° of 2θ, corresponding to [101] and [200] crystallographic planes of the TiO_2_ anatase phase, according to the PDF 00-064-0863 card. The low intensity and high broadness of the peaks for nanostructured TiO_2_ may be attributed to the presence of TiO_2_ crystals with the nanodomain size, the formation of which was promoted by the active species from the metabolites used, as reported by Logeswari et al., during the green syntheses of silver nanoparticles from organic aqueous extracts of different plants as well as *Solanum tricobatum, Syzygium cumini, Centella asiatica* and *Citrus sinensis*^44^. The crystal formation processes are based on the titania primary species nucleation principles, so their ordered growth in one direction is limited by the components presented in the metabolites. The crystal size of TiO_2_ was estimated by Scherrer´s equation (see Table 2). The results confirmed that all greenly synthesized TiO_2_ NPs were composed of relatively small crystals around 20 nm, while the reference TiO_2_ sample was characterized with a bigger crystal size (41 nm). These results correlate well with the SEM data.

**Table 2:** Summary of physicochemical properties of TiO_2_ nanoparticles prepared via green synthesis using different plant extracts.

TiO_2_ source	Crystallite size*, nm	Gap energy values**, eV	Specific surface area, m^2^/g	Pore volume, cm^3^/g
*Bougainvillea spectabilis*	20	3.20	113	0.21
*Moringa oleífera*	25	3.22	124	0.21
*Ricinus communis*	18	3.25	115	0.19
Commercial	41	3.29	7	0.007

*Calculated using the Scherrer´s equation on the peak at 2θ = 25.

**Estimated by the Tauc plot method.

**Figure 2:**
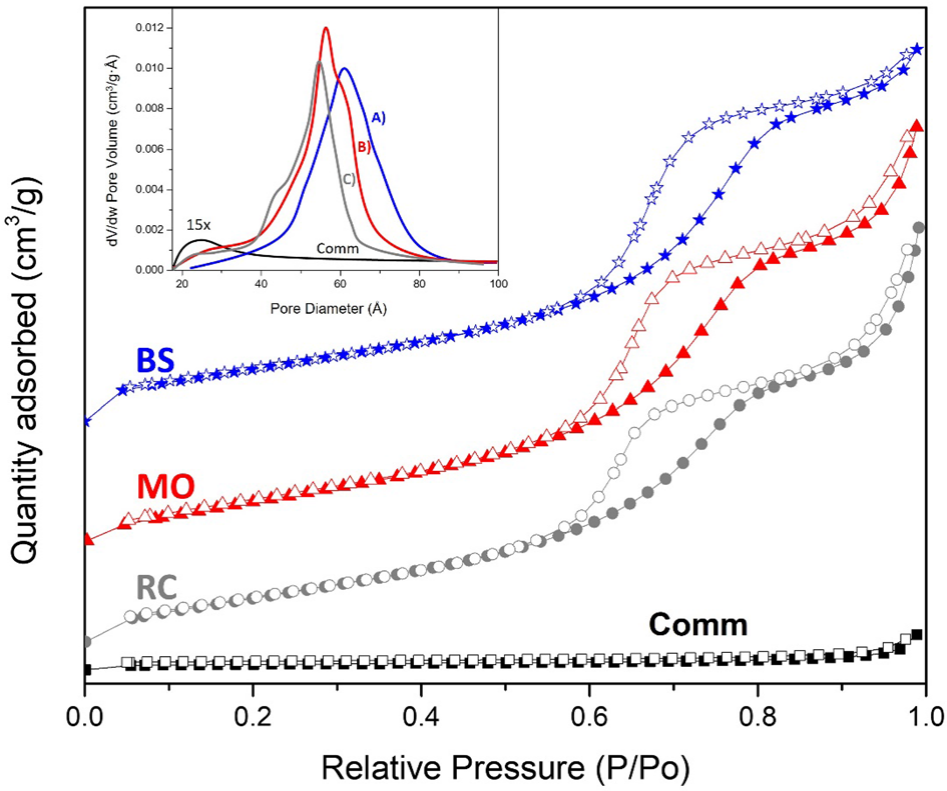
Isotherms of N2 adsorption for the greenly synthesized TiO2 samples using plant extracts of (A) RC, (**B**) MO and (**C**) BS and TiO2 (Comm) as a reference sample. The solid symbols indicate adsorption, while the open ones represent the desorption step. The inset presents the corresponding pore size distribution.

Figure 2 presents the adsorption–desorption isotherms for all samples. The isotherms for the greenly synthesized samples were characterized by a Type 4. At the same time, the comm TiO_2_ obtained isotherm was a Type III, commonly assigned to mesoporous and nonporous materials, according to the IUPAC classification. In addition, the hysteresis loop shape (H1) revealed the presence of cylindrical-like pore channels^31^. Therefore, thermal physisorption of N_2_ for prepared TiO_2_ NPs presented significant differences in the amount of gas adsorbed in comparison with the reference commercial sample (see Fig. 2). The latter coincided well with the remarkable improvement of the specific surface area values for green synthesized TiO_2_ NPs (see Table 2). The TiO_2_ NPs prepared by green synthesis were characterized with similar values of surface area (~ 120 m^2^/g) and pore volume (~ 0.21 m^3^/g); meanwhile, for the comm TiO_2_ sample, these values were lower (7 m^2^/g and 0.007 m^3^/g, respectively). Furthermore, applying the BJH model for the physisorption data displayed an average porous size of around 5.5 nm for all synthesized samples (Table 2). Note that the TiO_2_ samples prepared using RC and MO demonstrated two types of pores (4.2 and 6.3 nm, respectively). Thus, the applied green synthesis allowed the formation of relatively small TiO_2_ nanocrystals to form TiO_2_ NPs in the anatase phase with a remarkable enhancement of the surface area. Furthermore, the mesoporous nature of the greenly synthesized TiO_2_ NPs was revealed. Therefore, it may conclude that the green synthesis affects the textural properties of the prepared TiO_2_ NPs, which makes them functional for the proposed photocatalytic reaction because no mass transport limitations are expectable.

**Figure 3:**
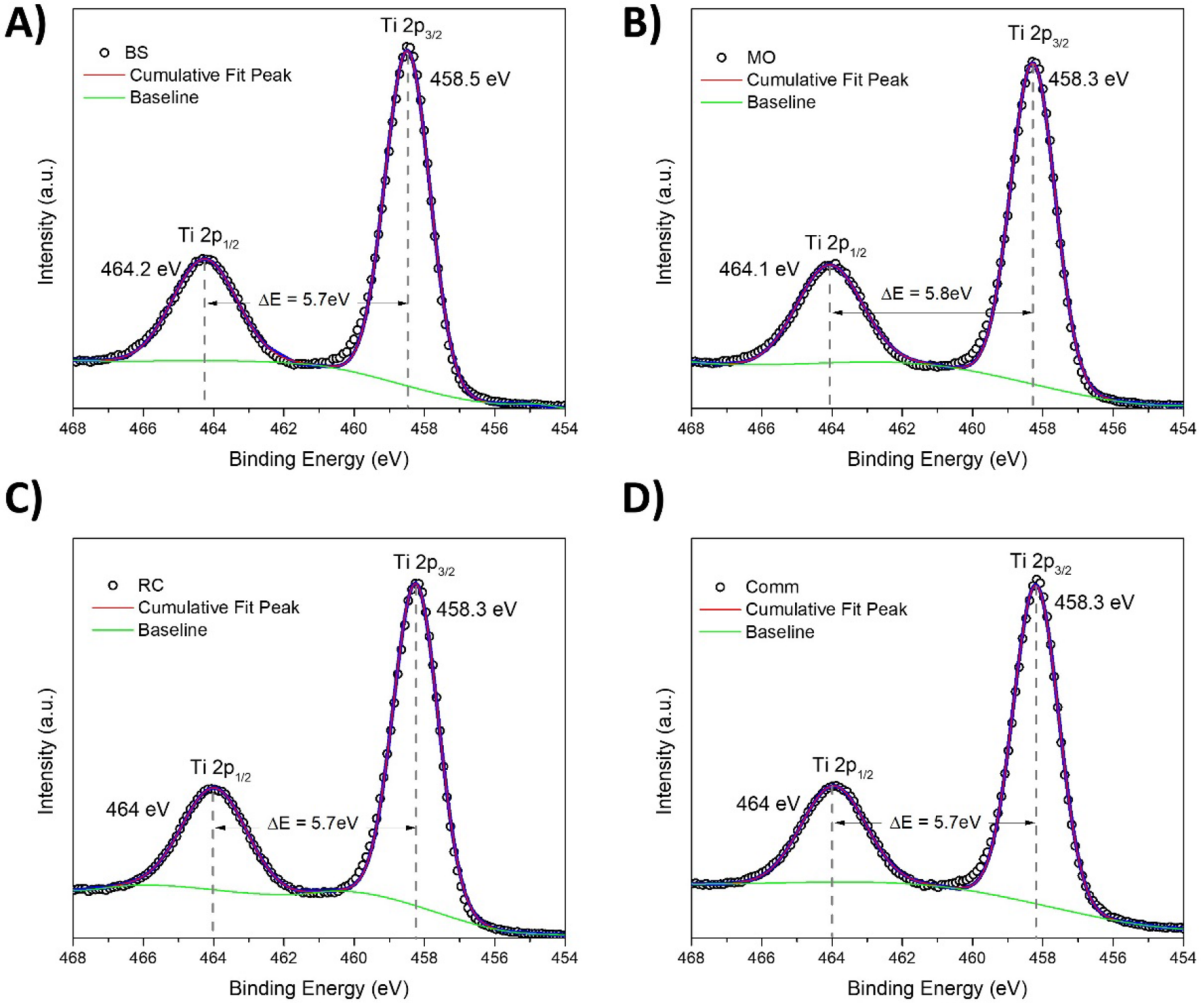
Ti2p XP spectra for the TiO_2_ samples prepared with the plant extract BS (**A**), MO (**B**) and RC (**D**) and the reference comm TiO_2_ (**D**). The circles represent the experimental data; the blue curves represent the Gaussian fits of the experimental data.

The high-resolution XP spectra of comm TiO_2_ (D) and prepared samples BC (A), MO(B) and RC (C)for the Ti2p region are presented in Fig. 3. For the TiO_2_ sample, the Ti2p spectrum is characterized by two spin–orbit components, Ti2p_3/2_ and Ti2p_1/2_, that are separated by 5.7 eV^45^, this signals exhibited several doublets attributed to different Ti oxidation states such as Ti^3+^ or Ti^4+^^46^. For all samples, the obtained Ti2p core level spectra presented two symmetric bands centered at binding energies of c.a. 458.5 and 464 eV assigned to Ti2p_3/2_ and Ti2p_1/2_, respectively, for the Ti^4+^ chemical state. Similar results are cited in the literature for TiO_2_ prepared via green methods^46–48^. Furthermore, a Ti^3+^ oxidation state is reported when surface defects are formed^46^ or dopants are incorporated into the TiO_2_ green synthesis procedure. In contrast, the Ti^4+^ oxidation state is found when only plant extracts are used^49,50^. Thus, it was confirmed that the maximum oxidation state of Ti for the greenly synthesized TiO_2_ NPs was reached. However, the plant extract did not affect the Ti chemical state achieved under green synthesis.

**Figure 4:**
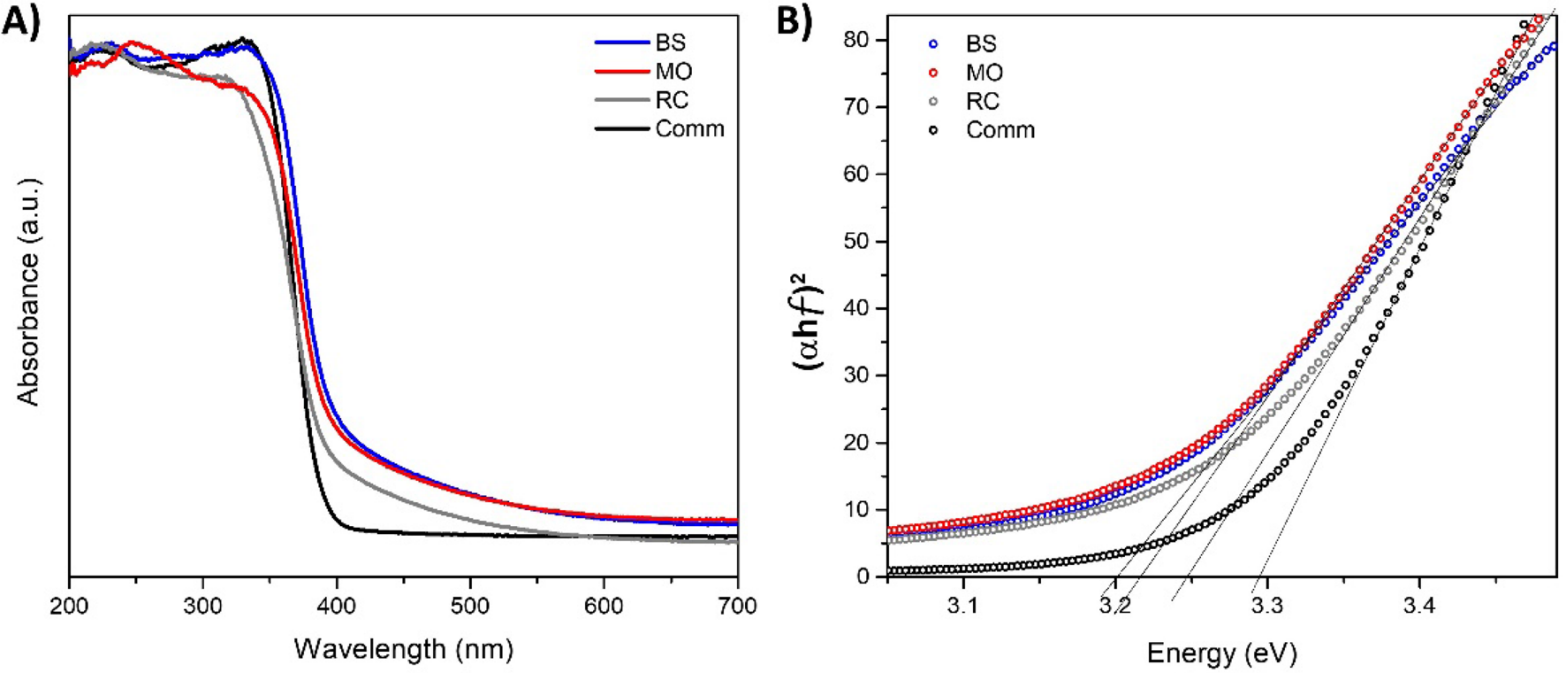
(**A**) UV–Vis absorption spectra of TiO_2_ samples prepared via green synthesis using extracts of BS, MO and RC. The UV–Vis spectrum of commercial TiO_2_ (Comm) is presented as a reference. (**B**) Estimation of band gap energies based on the Tauc plot method.

The UV–Vis absorption spectra of the synthesized samples were similar to those of the comm TiO_2_ (see Fig. 4A), characterized by an absorption edge in the near UV region due to the charge transfer between Ti and O. It is well-known that the band gap energy values are associated with the photocatalytic performance of titanium. The band gap energy for these materials was estimated from the UV–Vis absorption edge wavelength of the interband transition according to the Tauc plot method described in Ref.^51^ (see Fig. 4B). The band gap energy value for comm TiO_2_ was 3.29 eV, which is in good agreement with the literature data for the TiO_2_ oxide in the anatase phase (~ 3.30 eV)^52^. Meanwhile, the values obtained for the greenly synthesized TiO_2_ samples were 3.20, 3.22 and 3.25 eV, which were samples obtained from BS, MO and RC, respectively (see Table 2). These results agree with those reported for TiO_2_ NPs prepared via a green approach^13^. The decreased optical band gap for greenly synthesized TiO_2_ NPs makes it possible to propose that e- and h + pairs can be generated, promoting photocatalysis. Usually, synthesized TiO_2_ NPs are characterized by a decrease in band gap energy value because the structure formation is hardly influenced by the ordered aggregation of fine crystals, as was previously discussed. Instead, these fine crystals promoted the presence of surface defects that decreased the band gap energy value^51^. Therefore, the estimated band gap energy values were influenced by forming domains with several sizes promoted by the phytochemical components presented in the extract plant. The latter was previously confirmed by the crystal size calculations (see Table 2).

### Photocatalytic evaluation

#### Nejayote characterization

The composition analysis of nejayote revealed 32.5% of pentoses (ribose, arabinose, and xylose) and 47% of hexoses (glucose). Another 20.5% was attributed to other compounds such as lignin, inorganic matter (Ca, mainly), proteins, etc.

Table 3 shows the determination of elements in the solid sample of nejayote and ash (product of heat treatment of the nejayote solid sample). The results showed a high contribution of calcium and oxygen that´s characteristic of ¨calcium oxide¨, which is used for the ¨nixtamalization¨ process and the generation of nejayote (wastewater). In addition, it was observed that there was no carbon in the ash sample, which proves the absence of organic matter (polysaccharides and/or sugars).

**Table 3:** Elemental evaluation of organic and inorganic matter in the nejayote.

Sample	%C	%Ca	%Cl	%K	%O
Nejayote (solids)	36.61	48.95	0.46	1.46	12.54
Nejayote (ash)		35.24	1.21	7.93	53.44

#### Furfural production

**Figure 5:**
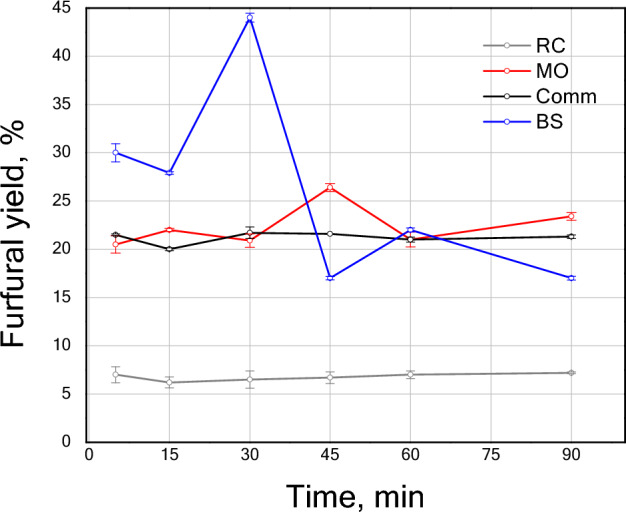
Furfural yield versus time from nejayote conversion using TiO_2_ comm and TiO_2_ sample prepared via green synthesis using extracts of BS, MO and RC at room temperature.

Figure 5 displays the furfural yields obtained from the nejayote photohydrolysis using the greenly synthesized TiO_2_ NPs and comm TiO_2_ as catalysts. All samples displayed some degree of furfural production during the 90 min of reaction monitoring. Figure S2 shows the proposed reaction pathway that lead to furfural production. The first step is the polysaccharide decomposition to form the xylose (pentose) that reacts further to form furfural; the newly formed furfural can react even further going through resinification, condensation or degradation. This furfural reactions lead to furfural loss^53^ that competes with the furfural production, causing instability that could explain the variations observed. Furfural concentration can be affected by:

1. The rate of polysaccharide decomposition to form xylose.
2. The rate of dehydration reaction of xylose to form furfural.
3. Furfural decomposition to form organic acids^54^.
4. The rate at which the furfural reacts with other molecules in the molecules in the reaction mixture; condensation reaction rate.
5. The rate at which the furfural reacts with other furfural molecules; resinification rate.

All reactions represented in Fig. S2 are affected by the reaction conditions and the photocatalytic properties of the catalyst.

Within the first 30 min the materials catalytic activity can be ordered as BS > MO≈comm TiO_2_ > RC. TiO_2_ NPs obtained using S-Met from BS displayed a 44% furfural yield. Since the feedstock employed was nejayote, instead of high-purity xylose, this result was unprecedented. Indeed, the furfural yield reached by the BS sample was 0.6 and 0.5 times that of the yield presented by Alonso et al.^9^ and Gallo et al.^55^, respectively, when xylose was used. Additionally, the reaction conditions in the present work were environmentally beneficial because (1) no extra water or energy to raise the temperature of the reactor was required^9,55^; and (2) no additional solvents such as GVL, toluene, THF, etc.^9–56^ were used.

The furfural production was very low for the RC sample, with a yield below 6% during the reaction. On the contrary, the MO and comm TiO_2_ samples demonstrated similar behavior for the furfural production with relatively constant product formation for MO the maximum yield (~ 25%) was detected at 45 min. From the data obtained, it can be inferred that the MO and comm TiO_2_ have a slower xylose hydration stage than that of the BS sample. As the BS sample reached the highest furfural concentration in a very early stage, this accelerated the undesired furfural reactions and prompted the depletion of xylose towards the end of the reaction time here studied^54^.

The low output of the furfural for the RC sample may be attributed to differences in the polysaccharides rupture (glycosidic bond) pathways^56,57^, which can produce a different ratio of xylose, arabinose, and ribose^56^. The latter may impact the product distribution in their subsequent hydrolysis. Furfural production is favored at high ratios of xylose, which has been described as a molecule with an isomerization process that leads to faster furfural production through xylulose and lyxose^58^. Additionally, nejayote contains relatively big molecules, such as polymers and proteins, that can affect the depolymerization and dehydration processes involved in furfural synthesis. From this perspective, the pore size and morphology of greenly synthesized TiO_2_ NPs may affect the diffusion of the molecules due to the nature of the feedstock.

Note that the performance of the MO sample is comparable with that presented for the comm TiO_2_. The comm TiO_2_, characterized by low surface area, mainly contributes to the external surface area. The latter may explain the lack of diffusional limitations of byproducts or impurities. In contrast, all greenly synthetized TiO_2_ NPs displayed a smaller particle size and higher surface area promoting the diffusion of the reactive species. However, the MO samples present the largest crystal size compared to the green synthesized BS and RC. The big crystal size accompanied by the small particle size could promote the formation of external active sites, favoring the photocatalytic activity of the material particles' external surfaces, leading to a similar behavior to that of the comm TiO_2_.

It was determined that the recovered catalyst for the recycling test absorbed organic and inorganic components from the nejayote. These components were identified by EDS and XRD after calcination; EDS revealed that Ca has deposited in the recovered material, ~ 5% Ca after reaction (Table S2). The XRD of the calcined sample also showed some carbon structure peaks that were not present in the fresh catalyst. The UV–Vis analysis of the recycled samples also showed a shift in the bandgap (from 3.2 to 3.06 eV) (Fig. S3). This is strong evidence of catalyst modification that could promote hemicellulose reactions that are detrimental for furfural production. Furfural yield reached 7.8% in 30 min using reused BS, which is 5.6 × the maximum yield obtained by BS in the first reaction (44%).

## Conclusions

The present work reports the effect of the plants' S-Met on the physicochemical properties and photocatalytic performance of the greenly synthesized TiO_2_ NPs. The S-Met extract from the BS plant to synthesize TiO_2_ NPs is used for the first time, the BS metabolites differ greatly RC and MO metabolites. When BS is used the crystal size and gap energy values decreased; meanwhile, the surface area increased up to 17 times in the greenly synthesized TiO_2_ NPs when compared to the comm TiO_2_. In this stydy we reported for the very first time the furfural production via the photohydrolisis of the nejayote, which is an unexplored residual biomass. The maximum furfural yield achieves 44% on the BS catalysts. The furfural yield reached by the greenly synthesized TiO_2_ NPs is comparable and 2 times superior to the reference comm TiO_2_, following the BS > MO≈comm TiO_2_ > RC trend. The intrinsic nejayote composition and the catalysts’ surface both interfere with the polysaccharide fragmentation through the glucoside bond breakage to form pentoses and their subsequent transformation into furfural. The nejayote photohydrolisis reaction on the greenly synthesized TiO_2_ NPs presents a comparable furfural yield to those reported when a high-purity xylose reagent is used.

The Chapala, México marshland region provides beneficial S-Met extracts to promote the synthesis of active, sustainable, and environmentally friendly catalysts. Furthermore, this work shows the potential to exploit a complex feedstock, promoting the lignocellulosic residual biomass valorization, to produce highly valuable compounds, such as furfural.

### Supplementary Information

Supplementary Information.

## Data Availability

The datasets generated and/or analysed during the current study are not publicly available due, but could be available from the corresponding author on reasonable request.
