# Supplementary material for: Green synthesis of TiO2 for furfural production by photohydrolysis of tortilla manufacturing waste
Source: Sci Rep. 2023 Sep 16;13:15355. doi: 10.1038/s41598-023-41529-z (PMC10505206; doi:10.1038/s41598-023-41529-z)
Supplement: Supplementary file 1 — Supplementary Information. [file 41598_2023_41529_MOESM1_ESM.docx]

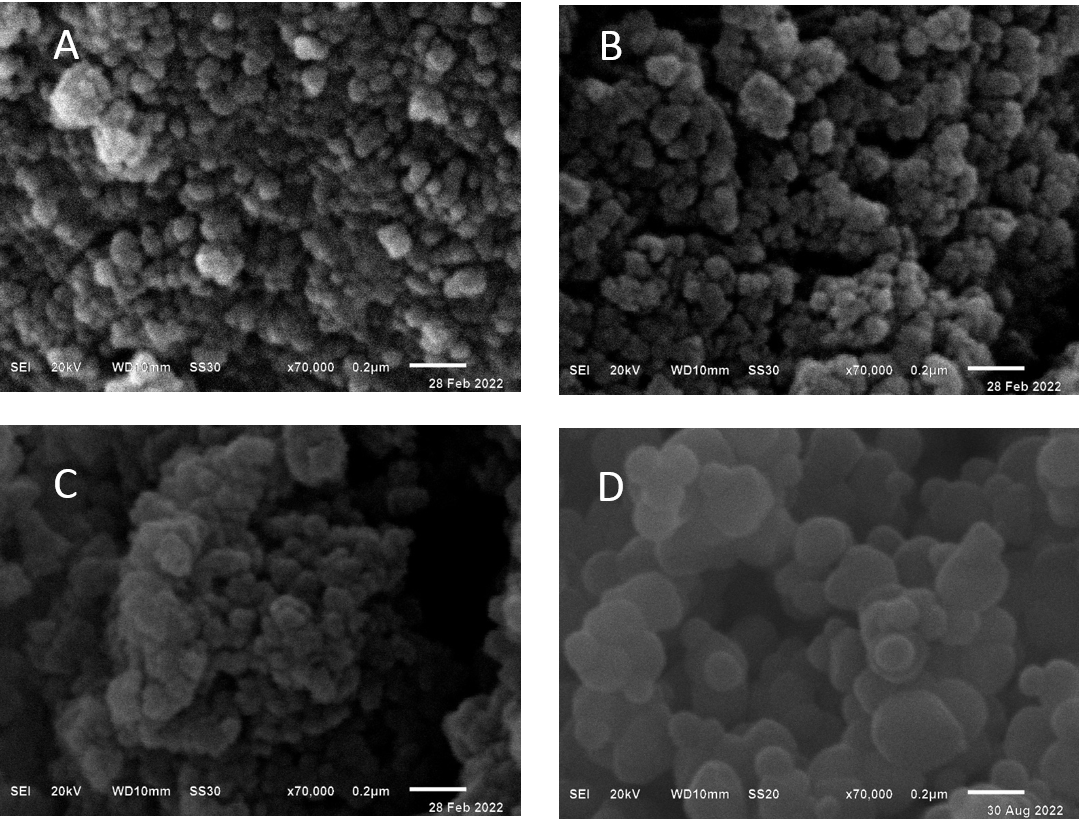


|  |  |
| --- | --- |
|  |  |

**Figure S1**. SEM micrographs of TiO_2_ samples obtained by Green synthesis with: A) *Bougainvillea spectabilis*, B) *Moringa oleífera*, C) *Ricinus communis* and D) TiO_2_ comm.


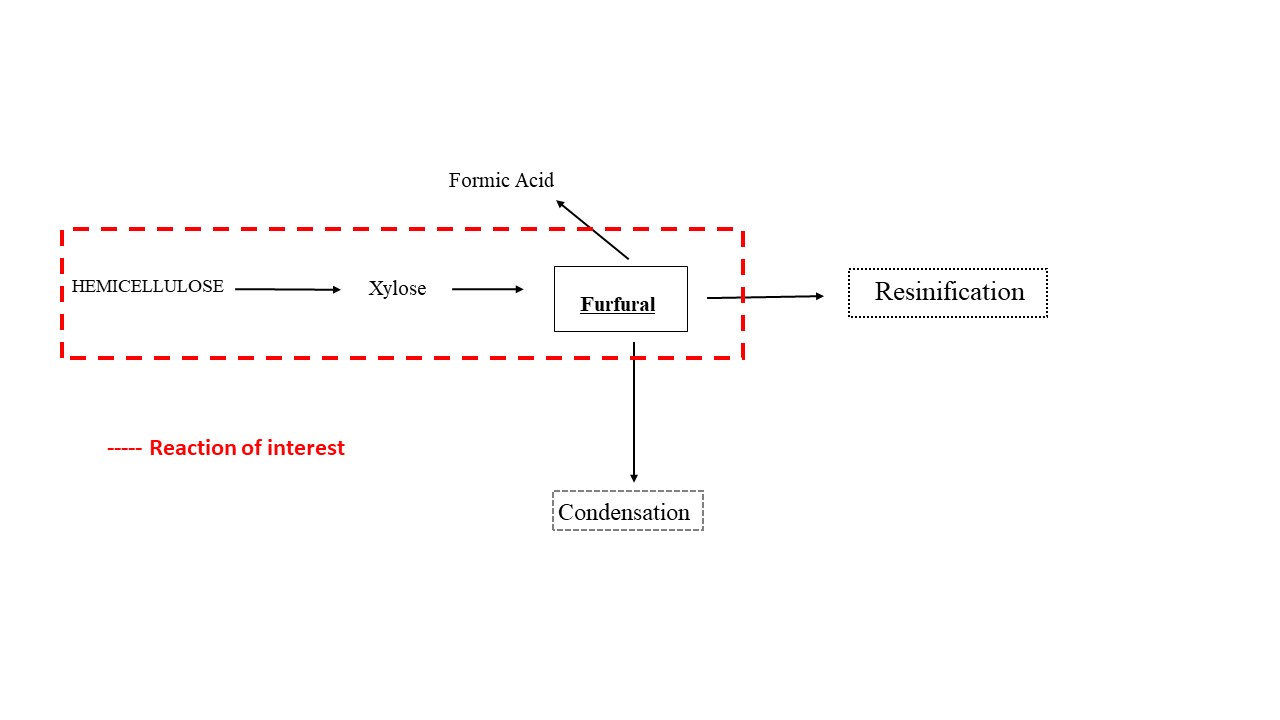


**Figure S2.** Reaction pathway of interest for furfural formation.

|  |  |
| --- | --- |

|  |  |
| --- | --- |
|  |  |

**Figure S3**. UV-Vis absorption spectra of TiO_2_ sample recycled (re-used) prepared via green synthesis using extracts of BS.

**Table S1**. Qualitative information of TiO_2_ samples.

| **TiO_2_ source** | **O, weight %** | **K, weight %** | **Ti, weight %** |
| --- | --- | --- | --- |
| *Bougainvillea spectabilis* | 41.87 | 0.49 | 57.65 |
| *Ricinus communis* | 55.05 | 0.42 | 44.53 |
| *Moringa oleífera* | 22.09 | 0.78 | 77.13 |
| Commercial | 50.85 | N.D.* | 49.15 |

**Table S2**. Qualitative information of TiO_2_ NPs post-photohydrolysis.

| **TiO_2_ source** | **O, weight %** | **K, weight %** | **Ti, weight %** | **Ca, weight %** |
| --- | --- | --- | --- | --- |
| *Bougainvillea spectabilis* | 19.35 | 1.37 | 72.90 | 4.78 |
